# Investigating relationship between water production and interfacial activity of γ-oryzanol, ethyl ferulate, and ferulic acid during peroxidation of bulk oil

**DOI:** 10.1038/s41598-021-96439-9

**Published:** 2021-08-23

**Authors:** Mohamad Reza Toorani, Mohammad-Taghi Golmakani

**Affiliations:** Department of Food Science and Technology, School of Agriculture, Shiraz University, 71441-65186 Shiraz, Iran

**Keywords:** Chemical physics, Chemical biology

## Abstract

In this study, lecithin (as a surfactant) was added to promote the inhibitory-mechanism of γ-oryzanol, ethyl-ferulate and ferulic acid (based on the interfacial phenomena) so as to inhibit the oxidation of stripped sunflower oil. Monitoring the amount of water production as a byproduct of oxidation showed that the water content of the lipid system increased remarkably through the oxidation progress. Lecithin enhanced the critical concentration of hydroperoxides in reverse micelles, compared to the basic state (14.8 vs. 9.2 mM), thereby improving the hydrogen-donating mechanism of antioxidants. The size of reverse micelles increased progressively during the oxidation, while two breakpoints were pointed out in the micelles growth, i.e. at the end of the initiation and the propagation phases. Based on the kinetic data, ferulic acid showed the highest antioxidant activity (23.4), compared to ethyl-ferulate (15.5) and γ-oryzanol (13.7). Generally, lecithin enhanced antioxidant activity (~ 65%) by improving the interfacial performance of antioxidants.

## Introduction

Oxidation reaction is one of the main concerns in reducing the quality and deterioration of vegetable oils. The process of oxidation occurs more rapidly in oils with polyunsaturated fatty acids and comprises three consecutive periods (i.e. initiation, propagation, and termination phases). The process involves the production of hydroperoxides (ROOHs) which is of particular importance as precursors of all oxidation products^1^. Monitoring the accumulation of ROOHs during different stages of oxidation can provide valuable information for researchers about events during different stages and transfer of phases. Undoubtedly, such an information can contribute to the inhibition of oxidative reactions.

The ROOHs production in the initiation stage of oxidation is a type of zero-order reaction^2^. This process continues until the point where suddenly the slope of ROOHs production increases dramatically. Known as the ROOH_IP_, the said point coincides with the phase transition from the initiation phase to the propagation phase^3^. By passing this stage, the slope of ROOHs production continues to increase until it reaches its highest level in the middle of the propagation phase. From this point onwards, known as the turning point or the maximum rate (M_R_), the decomposition reaction of ROOHs begins^4^. The occurrence of this reaction as an equilibrium reaction continues until it reaches a balance between production and decomposition of ROOHs^5^. This irreversible point is considered as ROOH_max_ (maximum achievable concentration) and is associated with surpassing the decomposition rate of ROOHs, compared to their formation rate^1,4^. Such behavior has good potential to be interpreted by a sigmoidal kinetic model in which several important indices exist. One of these indices is the rate constant of pseudo-first-order (*k*_f_), known as a measure of the formation of ROOHs (or oxidizability of lipid systems) in the propagation phase. Another criterion is the rate constant of pseudo-second-order (*k*_d_) which represents the decomposition of ROOHs in the propagation phase^5,6^. Valuable information can result from combining the indices of the sigmoidal model and by generalizing them to physicochemical events that occur at the oxidation phase transfer.

Ferulic acid (FRA) is a well-known antioxidant in many products of herbal origin. In the benzene ring of this hydroxycinnamic acid, methoxy and hydroxyl groups occur simultaneously and adjacent to each other. The methoxy group in the benzene ring can make an intramolecular hydrogen bond by creating a hydrogen bridge with its nearby OH-group^7^. This reaction often occurs in nonpolar environments, causing the hydrogen donating mechanism (by hydroxyl group) to become somewhat inactive^8^. However, after hydrogen separation, the presence of the methoxy group can stabilize the remaining electrons, as this happens by their delocalization, and facilities the mechanism of electron transfer^9^. This paradoxical behavior of the methoxy group causes performative changes in antioxidant mechanisms according to their functional environment. Phytosteryl ferulate or gamma oryzanol (GOR) is a renown natural antioxidant that originates from rice bran oil^10^. This antioxidant has higher solubility due to the low ratio of hydrophilic/hydrophobic moieties, compared to ferulic acid in the lipid substrate. Ethyl ferulate (EFR) is also derived from ferulic acid with more lipophilic characteristics and less antioxidant capacity. Naturally, it is isolated from giant fennel^11^.

Vegetable oils contain small amounts of water, although they originate from oil seeds and through refining processes and surfactants that may exist naturally in the source (such as mono- or di-acylglycerols and phospholipids) or which could be produced during the oxidation process (e.g. ROOHs, alcohols, aldehydes, and ketones). In the presence of water, these surface-active agents can create reverse micelles or lamellar structures by reducing interfacial tension. Thus, vegetable oils contain regular physical structures and, as a matter of fact, oxidation reactions occur in these microreactors^12,13^. Molecules with higher polarity, compared to the polarity of triacylglycerols (such as antioxidants or free radicals), exhibit a greater tendency to migrate to water–oil interfaces where inhibitory reactions have a high chance of happening^14,15^. As oxidation progresses further and adds to the production of ROOHs, the number of micelles and their size increase until they reach a critical micellar concentration (CMC), followed by an eventual collapse. This point is exactly equal to ROOH_IP_ wherein the oxidation process enters the propagation phase by releasing a large volume of ROOHs throughout the environment and optimizes collisions between free radicals^16^.

Lecithin (LEC) as a phospholipid is an amphiphilic compound that can protect vegetable oils against oxidation. In a relevant literature review, various roles of performance have been suggested for this compound in vegetable oils and in preventing their oxidation. Several of these performative roles include the regeneration of primary antioxidants, metal chelating and the establishment of an oxygen barrier between the oil and air interfaces^15,17–19^. Another important performance of LEC can be seen in relation to its role in supporting the formation of reverse micelles during the oxidation process. Considering the fact that phospholipids can markedly reduce interfacial tension, the number and size of microreactors are likely to increase significantly in the presence of specialized surfactants. As a result, there can be an increase in the acceptance capacity of ROOHs in these structures. Since, relatively polar antioxidants are precisely located in the interfaces of micro-micelles^13^, more interactions can occur between antioxidant molecules and ROOHs. This physical role of LEC can lead to a delay in achieving CMC and to an increase in the duration of the induction period (IP). However, less attention has been given to a part of the antioxidant that comes in the contact area between oil/storage-container and air/oil (due to the difference in the polarity)^20^. The presence of surface-active agents is assumed to excite the movement of this part of antioxidant molecules into the water–oil interface by increasing the number of microreactors.

In this regard, the present study aimed to investigate the antioxidant activity of FRA and its derivatives with different alkyl chains (EFR and GOR) in the presence of LEC to elucidate the effects of interfacial phenomena on bulk oil peroxidation. Furthermore, various oxidation indices pertained to the initiation and the propagation phases were evaluated to clarify the details of physicochemical events that occurred during the oxidation process.

## Materials and methods

### Materials

Refined sunflower oil was purchased from Golbarg-e-Baharan Company (Karaj) as an oxidative substrate. The GOR (CAS No. 11042-64-1) was purchased from TCI Chemicals Company (Tokyo, Japan). Meanwhile, FRA (CAS No. 1135-24-6), EFR (CAS No. 4046-02-0), and LEC (1-oleoyl-2-linoleoyl-sn-glycero-3-phosphocholine) (CAS No. 8002-43-5) were purchased from Sigma Aldrich (St. Louis, MO). All other solvents, chemicals and standard markers were purchased from Merck (Darmstadt, Germany) and Sigma Aldrich companies.

### Oil purification process

The bulk oils contained minor components that may interfere with the performance of antioxidants or may affect the oxidation process. To eliminate these components, the purification process was performed by an adsorption chromatography column. To this end, two-glass column series were used (columns size, 36 cm height and 29 mm internal diameter). Each column comprised three layers of adsorbent (from the top layer to the bottom; 5 g of activated carbon, 30 g of silica gel, and 50 g of aluminum oxide 60). All sorbents were activated at 180 °C for 4 h. Almost 120 g of each oil was added to the first column slowly and gradually. A vacuum pump with high pressure was utilized to facilitate oil withdrawal from the chromatography column. The contents of the output from the first column was transferred to the second column and this operation was repeated once more. The purified samples were maintained at − 18 °C (for a maximum period of two weeks) and the headspace was filled with nitrogen. According to previous research, this method can remove or significantly diminish tocopherols, phenolic compounds and metal elements^21^.

### CMC of LEC in sunflower oil

Tetracyanoquinodimethane (TCNQ) was used as a reagent to measure the CMC of LEC. For this purpose, a blend of TCNQ and purified sunflower oil (with the ratio of 1:1, v/w) was made to contain 0.015–0.2% LEC. This blend was vortexed for 5 h by a magnetic stirrer at ambient temperature. To remove the TCNQ excess, the blend was centrifuged at 2000×*g* for 15 min. The supernatant was carefully collected and the absorbance was measured at 480 nm by a spectrophotometer. The standard curve was plotted using the LEC concentration vs. TCNQ absorption, and the tangent method was employed to calculate coordinates that demonstrated the CMC of LEC^22^.

### Preparation of inhibited peroxidation

The peroxidation process of sunflower oil involved using a dry oven at 60 °C. Briefly, 6 g of purified oil was added to a Petri dish (6 cm diameter) to provide a thin layer of oil. This condition causes the peroxidation rate not to be affected by the oxygen concentration. To prepare inhibited peroxidation, 0.33 mM of each antioxidant was dissolved in 1 mL of acetone, and was added to the lipid substrate. The added solvent was eventually eliminated by nitrogen gas. To provide samples containing LEC, 6.60 mM of LEC (molecular weight: 758.1 g mol^−1^) (ratio 1:10 w/v) was dissolved in ethyl acetate for 1 h at 40 °C by a magnetic thermo-stirrer. Then, the purified oil was slowly added to the cooled solution and the stirring process remained at ambient temperature for 10 min. In the next step, the added solvent was removed by a rotary evaporator. Thus, the lipid substrate containing 500 µg mL^−1^ of LEC was prepared^23^. Then, 0.33 mM of each antioxidant was separately added to the different Petri dishes, containing 6 g of lipid substrate and LEC.

### Log P

The partition coefficients of the antioxidants under study, including their solubility ratio in a nonpolar to polar environment as log *P*, were computed using ChemDraw software (version 16 Professional; PerkinElmer, Waltham, MA, USA).

### Monitoring the accumulation of ROOHs

This process was carried out by sampling treatments under the peroxidation at certain time intervals. Then, the peroxide value was measured by a spectrophotometer according to Shanta and Decker (1994). For this purpose, regarding the peroxidation progress between 0.001 and 0.3 g of oil sample, the oil was weighed in 15 mL test tubes. Then, 9.8 mL chloroform–methanol (7:3, v/v) was added to the oil samples. Fifty µL of ammonium thiocyanate aqueous solution (30%, w/v) was added to the oil sample and shaked for 5 s. The next stage involved mixing 50 µL Iron (II) chloride solution ([0.25 g FeSO_4_·7H_2_O dissolved in 25 mL H_2_O] + [0.2 g barium chloride dehydrate dissolved in 25 mL H_2_O] + 1 mL HCl 10 N, and then the resultant solution was filtered to remove barium sulphate deposits). After 5 min, the sample absorption was determined at 500 nm^24^. Eventually, the results were reported based on milliequivalent of oxygen per kg of oil (meq kg^−1^) or molarity (1 meq kg^−1^ = 0.504 mM)^25^.

### Kinetic parameters

**Figure 1:**
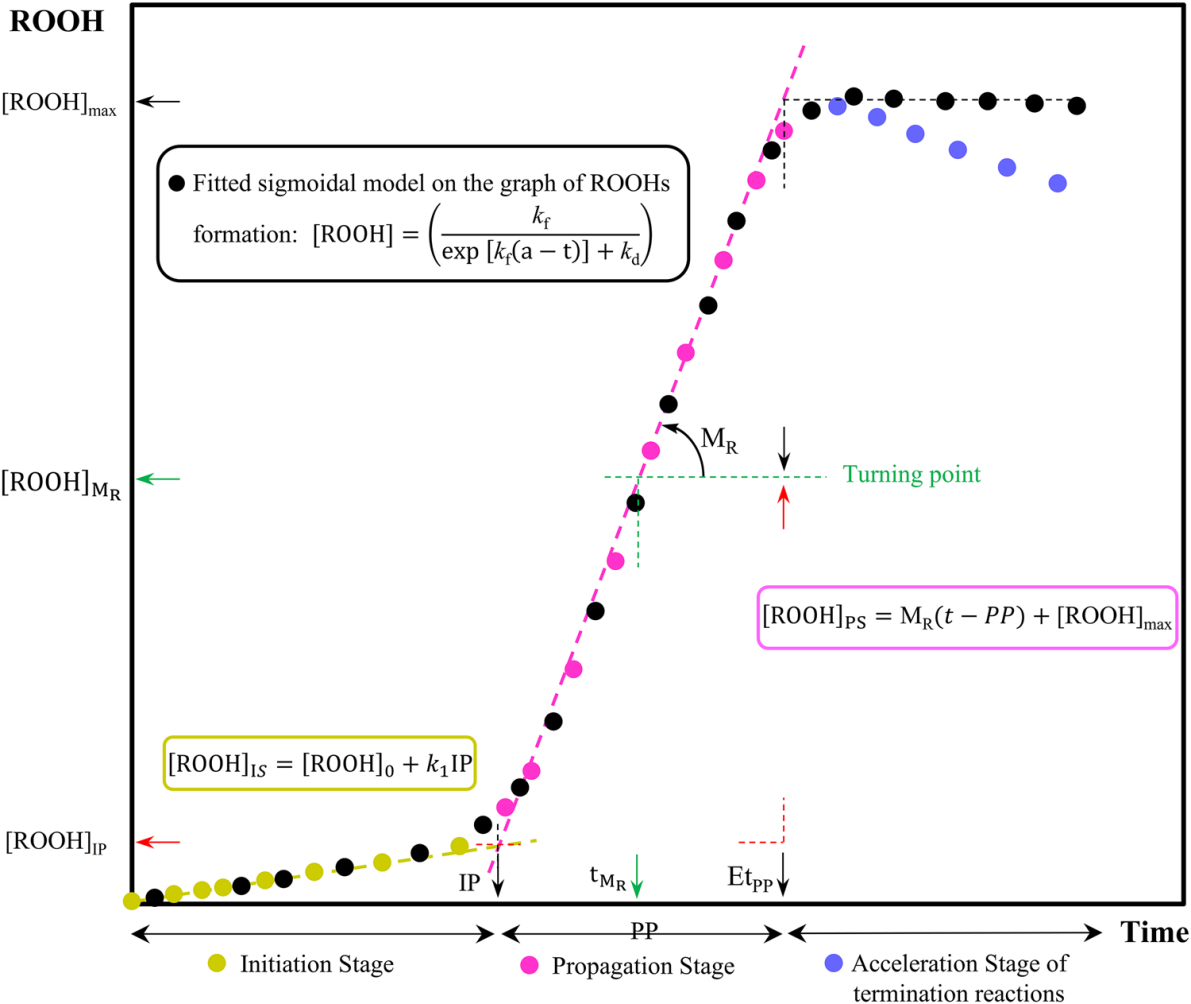
Schematic curve of hydroperoxides (ROOHs) production and a guide of calculated kinetic points. *Et*_*PP*_ end time of the termination phase, *IP* induction period, *k*_*f*_ rate constant of ROOHs formation at the propagation phase, *k*_*d*_ rate constant of ROOHs decomposition at the propagation phase, *k*_*1*_ rate constant of the initiation phase, *M*_*R*_ maximum rate of ROOHs formation in the propagation phase, *PP* propagation period, *ROOH* ROOHs concentration, *[ROOH]*_*IP*_ ROOHs concentration at IP point, *[ROOH]*_*max*_ maximum concentration of produced ROOHs, ${\left[ROOH\right]}_{{M}_{R}}$ ROOHs concentration at the point of the maximum rate of ROOHs formation (or turning point), ${t}_{{M}_{R}}$ occurrence time of maximum rate of ROOHs formation (or turning point).

As shown in Fig. 1, various kinetic parameters were obtained by plotting changes in ROOHs vs. time. Several equations were used in calculating these parameters, according to the following^2,6^:

The oxidation reaction rate in the initiation phase can be expressed by Eq. (1):

1 $$\frac{\mathrm{d}[\mathrm{ROOH}]}{\mathrm{dt}} ={k}_{1}$$

where *k*_1_ is the rate constant of the initiation phase. Equation (2) is obtained by integrating Eq. (1) vs. the limited time from zero time to IP point, and a concentration range from [ROOH]_0_ (ROOHs amount at t = 0) to [ROOH]_IP_:

2 $${[\mathrm{ROOH}]}_{\mathrm{IP}}={[\mathrm{ROOH}]}_{0}+{k}_{1}\mathrm{IP}$$

As mentioned earlier, to evaluate the behavior of vegetable oils against oxidation and, particularly, in the presence of antioxidants, a combination model was employed according to Eq. (3). Based on the pseudo-first-order reaction, the formation rate of ROOHs in the propagation phase (*k*_f_) were expressed by Eq. (3):

3 $$\frac{\mathrm{d}[\mathrm{ROOH}]}{\mathrm{dt}} ={k}_{\mathrm{f}}[\mathrm{ROOH}]$$

after integration:

4 $$[\mathrm{ROOH}]=\mathrm{exp }({k}_{\mathrm{f}}\mathrm{t}-{\mathrm{a}}_{\mathrm{f}})$$

where a_f_ is the integration constant. Also, Eq. (5) finds rate constant of the ROOHs decomposition (*k*_d_) in the pseudo-second-order reaction:

5 $$-\left(\frac{\mathrm{d}[\mathrm{ROOH}]}{\mathrm{dt}}\right)={k}_{\mathrm{d}}{[\mathrm{ROOH}]}^{2}$$

after integration:

6 $$\left[\mathrm{ROOH}\right]= \frac{1}{{k}_{\mathrm{d}}\mathrm{t}-{\mathrm{a}}_{\mathrm{d}}}$$

where a_d_ is integration constant. By merging Eqs. (3) and (5), we have:

7 $$\frac{\mathrm{d}[\mathrm{ROOH}]}{dt}={k}_{\mathrm{f}}[\mathrm{ROOH}]-{k}_{\mathrm{d}}{[\mathrm{ROOH}]}^{2}$$

the integration of Eq. (7) gives:

8 $$[\mathrm{ROOH}]=\frac{{\mathrm{k}}_{\mathrm{f}}}{\mathrm{exp}[{\mathrm{k}}_{\mathrm{f}}(\mathrm{a}-\mathrm{t})]+{\mathrm{k}}_{\mathrm{d}}}$$

where a is an overall integration constant. Also, [ROOH]_max_ is calculable by the following equation:

9 $${[\mathrm{ROOH}]}_{\mathrm{max}}={\mathrm{lim}}_{\mathrm{t}\to \infty }\left(\frac{{\mathrm{k}}_{\mathrm{f}}}{\mathrm{exp}[{\mathrm{k}}_{\mathrm{f}}(\mathrm{a}-\mathrm{t})]+{\mathrm{k}}_{\mathrm{d}}}\right)$$

Equation (8) is an empirical sigmoidal model that can be used for predicting the general trend of oxidation reaction by having a turning point in the middle of the propagation phase. The maximum achievable rate as M_R_ in this point can be obtained by Eq. (10):

10 $${\mathrm{M}}_{\mathrm{R}}={\left(\frac{\mathrm{d}[\mathrm{ROOH}]}{\mathrm{dt}}\right)}_{\mathrm{max}}=\frac{{{k}_{\mathrm{f}}}^{2}}{4{\mathrm{k}}_{\mathrm{d}}}$$

The normalized form (${{\mathrm{N}}_{\mathrm{M}}}_{\mathrm{R}}$) of the M_R_ can be obtained by Eq. (11):

11 $${{\mathrm{N}}_{\mathrm{M}}}_{\mathrm{R}}=\frac{{\mathrm{M}}_{\mathrm{R}}}{{[\mathrm{ROOH}]}_{\mathrm{max}}}$$

The coordinates of the turning point are calculated by the following equations:

12 $${{\mathrm{t}}_{\mathrm{M}}}_{\mathrm{R}}=\frac{{k}_{\mathrm{f}}\mathrm{a}-\mathrm{ln}{k}_{\mathrm{d}}}{{k}_{\mathrm{f}}}$$

13 $${{[\mathrm{ROOH}]}_{\mathrm{M}}}_{\mathrm{R}}=\frac{{k}_{\mathrm{f}}}{2{k}_{\mathrm{d}}}$$

The x-coordinate of the IP point in the combination model can be obtained by integrating Eq. (2) with Eq. (8) by the following equation:

14 $$\mathrm{IP}=\frac{{k}_{\mathrm{f}}(2-{k}_{\mathrm{f}}\mathrm{a}+\mathrm{ln}{k}_{\mathrm{d}})-4{[\mathrm{ROOH}]}_{0}{k}_{\mathrm{d}}}{4{k}_{1}{k}_{\mathrm{d}}-{{k}_{\mathrm{f}}}^{2}}$$

The end time of the propagation phase (Et_PP_) and propagation period (PP) are calculated by the following equations:

15 $${\mathrm{Et}}_{\mathrm{PP}}=\frac{4{k}_{\mathrm{d}}{\mathrm{M}}_{\mathrm{R}}-{k}_{\mathrm{f}}{{\mathrm{N}}_{\mathrm{M}}}_{\mathrm{R}}(2-{k}_{\mathrm{f}}\mathrm{a}+\mathrm{ln}{k}_{\mathrm{d}})}{4{k}_{\mathrm{d}}{\mathrm{M}}_{\mathrm{R}}{{\mathrm{N}}_{\mathrm{M}}}_{\mathrm{R}}}$$

16 $$\mathrm{PP}={\mathrm{Et}}_{\mathrm{PP}}-\mathrm{IP}$$

Antioxidant effectiveness (*E*) measured by Eq. (17):

17 $$E=\boldsymbol{ }\frac{{\mathrm{IP}}_{A}}{{\mathrm{IP}}_{\mathrm{C}}}$$

where IP_C_ and IP_A_ are the IPs in the absence and the presence of the antioxidant, respectively. The ratio of the oxidation rate (*R*_or_) is obtained as a measure of antioxidant strength (1/*R*_or_), according to the following equation:

18 $${R}_{or}=\boldsymbol{ }\frac{{{k}_{1}}_{\mathrm{A}}}{{{k}_{1}}_{\mathrm{C}}}$$

where *k*_1A_ and *k*_1C_ are the initiation rate constants of peroxidation in the presence and the absence of the antioxidant, respectively. By unifying the indices of Eqs. (17) and (18), the antioxidant activity was calculated according to Eq. (19):

19 $$A= \frac{E}{{R}_{or}}$$

The oxidation resistance in the initiation phase (*O*_*R*_) and synergistic effect (SE) of LEC was calculated by the following equations:

20 $${O}_{R}= \frac{IP}{{k}_{1}}$$

21 $$SE (\%)= \left(1-\frac{{{O}_{R}}_{A}+{{O}_{R}}_{L}-2{{O}_{R}}_{C}}{2({{O}_{R}}_{A+L}-{{O}_{R}}_{C})}\right)\times 100$$

where ${{O}_{R}}_{A}$,${{O}_{R}}_{L}$, ${{O}_{R}}_{C}$, and ${{O}_{R}}_{A+L}$ are oxidation sensitivity parameters of the antioxidant per se, LEC per se, control, and antioxidant + LEC, respectively.

### Carbonyl value

Carbonyl value (CV; μmol of 2,4-decadienal per gram of oil) was measured using 2-propanol purified by sodium borohydride^26^. For this purpose, a mixture (10 mL) of oil sample (0.04–1.0 g) was prepared using 2-propanol containing triphenylphosphine (0.4 mg mL^−1^). Then, 1 mL of the mixture was placed in a 15-mL test tube and mixed with 1 mL of 2,4 dinitrophenylhydrazine (DNPH) solution (50 mg of DNPH in 100 mL of 2-propanol containing 3.5 mL of HCl 37%). The test tube was stoppered and heated for 15 min at 40 °C. After cooling in the water bath, 8 mL of KOH solution (2%) were added. Centrifugation (2200×*g*) was performed for 4 min at room temperature. The absorbance of the supernatant was measured at 420 nm against 2-propanol as blank. A calibration curve of 2,4-decadienal in 2-propanol (50–500 μM) was prepared.

### Water content

The amounts of water being produced during the peroxidation process were measured by a Karl Fischer titrator device (KF Titrando, Metrohm, Herisau, Switzerland) in accordance with the manufacturer’s guidelines.

### Particle size

The changes in size and distribution of particles with peroxidation progress were analyzed by dynamic light scattering (DLS) (SZ-100 nanopartica series, Horiba Ltd., Kyoto, Japan) at a scattering angle of 173° and 25 °C.

### Statistical analysis

All tests were performed in three independent experiments and the results entered the analysis of variance. Statistical and regression analyses were performed using SPSS, CurveExpert, and Microsoft Office Excel software. Significant differences among the mean values were determined by Duncan’s multiple range test (*P* < 0.05).

## Results and discussion

### Evaluating primary kinetic parameters

**Figure 2:**
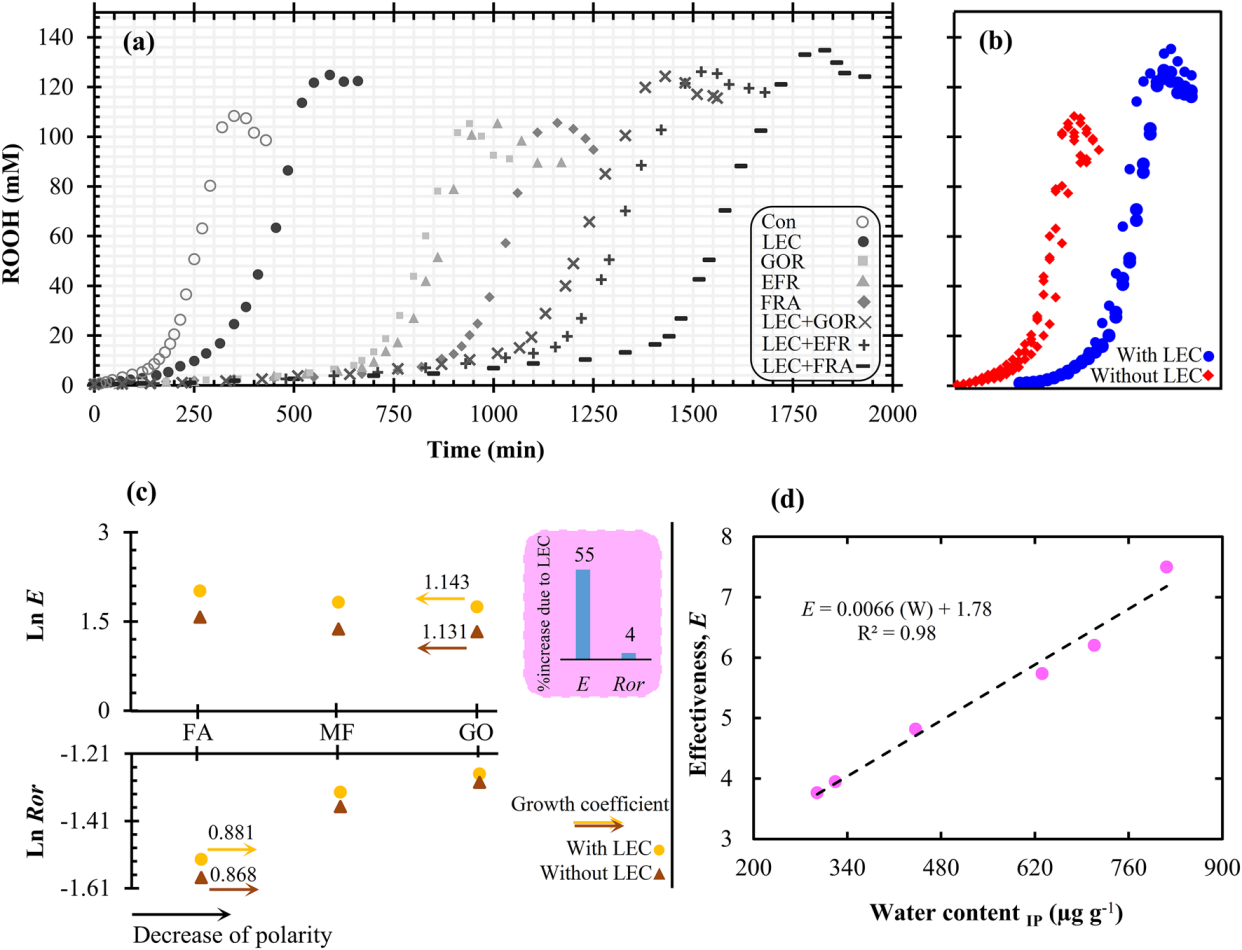
(**a**) Sigmoidal curve of hydroperoxides (ROOHs) accumulation in the peroxidation of stripped sunflower oil (Control) containing lecithin (LEC), ferulic acid (FRA), ethyl ferulate (EFR), γ-oryzanol (GOR) and their combinations at 60 °C, (**b**) comparison of graphs of ROOHs production in the presence or absence of lecithin, (**c**) displaying variations in the kinetic parameters of effectiveness (*E*) and the ratio of oxidation rate (*R*_or_) of antioxidants in the presence and absence of lecithin, and (**d**) the relationship between the effectiveness parameter of antioxidants and water content at the IP point.

The predicted sigmoidal model fitted well on the curve of ROOHs production and distinguished the different phases of the oxidation process (R^2^ ≥ 0.98). As shown in Fig. 2a, the longest duration of the initiation phase was recorded in samples containing LEC. Among them, the highest level was found in the FRA, followed by EFR and GOR. The exact values of IPs were listed (Table 1). Remarkable differences were observed in the performance of the antioxidants under study, although their phenolic rings were similar while being directly involved in displaying the antioxidant activity. Evaluating the *E* parameter, as a symbol to introduce the hydrogen donating mechanism^27,28^, revealed a significant increase in this factor. These results indicate the participation of antioxidant molecules in chain termination reaction as shown in the following equation^2^:

22 $$ {\text{AH}}\, + \,{\text{ROO}}^{ \bullet } \to \,{\text{ROOH}} + \,{\text{A}}^{ \bullet } $$

**Table 1:** Kinetic parameters related to the initiation, propagation, and termination phases of the stripped sunflower oil peroxidation (Control) containing lecithin (LEC), ferulic acid (FRA), ethyl ferulate (EFR), γ-oryzanol (GOR), and their combinations at 60 °C.

Sample	Kinetic parameters related to initiation and propagation phases								
	IP^A^	*E*^B^	*k*_1_ (× 10^2^)^C^	*R*_or_^D^	*A*^E^	ROOH_IP_^F^	PP^G^	*k*_*f*_ (× 10^2^)^H^	*k*_*d*_ (× 10^4^)^I^
Control	194 ± 2^h^*	–	9.00 ± 0.05^a^	–	–	18.2 ± 0.3^b^	127 ± 5^d^	2.87 ± 0.01^a^	1.35 ± 0.01^a^
LEC	352 ± 4^g^	1.82 ± 0.07^g^	8.15 ± 0.03^b^	0.91 ± 0.00^a^	2.0 ± 0.2^f^	29.4 ± 0.3^a^	210 ± 9^b^	1.69 ± 0.02^d^	0.64 ± 0.04^d^
FRA	934 ± 9^d^	4.82 ± 0.03^d^	1.85 ± 0.02^h^	0.21 ± 0.00^g^	23.4 ± 0.5^b^	18.1 ± 0.2^b^	173 ± 7^c^	2.11 ± 0.00^c^	1.02 ± 0.01^c^
EFR	765 ± 7^e^	3.95 ± 0.03^e^	2.29 ± 0.01^f^	0.25 ± 0.00^e^	15.5 ± 0.2^d^	18.3 ± 0.5^b^	168 ± 10^c^	2.16 ± 0.01^b^	1.11 ± 0.00^b^
GOR	730 ± 5^f^	3.77 ± 0.04^f^	2.46 ± 0.00^d^	0.27 ± 0.00^c^	13.7 ± 0.2^e^	18.7 ± 0.3^b^	167 ± 4^c^	2.16 ± 0.00^b^	1.08 ± 0.01^b^
LEC + FRA	1453 ± 19^a^	7.50 ± 0.07^a^	1.96 ± 0.01^g^	0.22 ± 0.00^f^	34.4 ± 0.6^a^	29.2 ± 0.2^a^	269 ± 12^a^	1.32 ± 0.01^f^	0.50 ± 0.00^f^
LEC + EFR	1202 ± 10^b^	6.20 ± 0.04^b^	2.39 ± 0.01^e^	0.27 ± 0.00^d^	23.3 ± 0.3^b^	29.5 ± 0.5^a^	251 ± 5^a^	1.40 ± 0.00^e^	0.56 ± 0.01^e^
LEC + GOR	1112 ± 8^c^	5.74 ± 0.03^c^	2.53 ± 0.01^c^	0.28 ± 0.00^b^	20.4 ± 0.1^c^	28.9 ± 0.4^a^	256 ± 9^a^	1.38 ± 0.00^e^	0.56 ± 0.00^e^

Sample	Continues kinetic parameters								
	[ROOH]_max_^J^	M_R_^K^	${{\mathrm{N}}_{\mathrm{M}}}_{\mathrm{R}}$(× 10^3^)^L^	${\left[\mathrm{ROOH}\right]}_{{\mathrm{M}}_{\mathrm{R}}}$^M^	${\mathrm{t}}_{{\mathrm{M}}_{\mathrm{R}}}$^N^	a^O^	Et_PP_^P^	*O*_*R*_ (× 10^3^)^Q^	${\mathrm{CV}}_{{\mathrm{M}}_{\mathrm{R}}}$^R^
Control	214 ± 8^c^*	1.53 ± 0.02^a^	7.18 ± 0.02^a^	107 ± 2^b^	251 ± 5^h^	− 59 ± 2^e^	321 ± 7^h^	2.15 ± 0.2^g^	55.6 ± 0.4^a^
LEC	263 ± 11^ab^	1.11 ± 0.00^b^	4.23 ± 0.00^d^	131 ± 5^a^	444 ± 9^g^	− 126 ± 3^f^	562 ± 11^g^	4.32 ± 0.1^f^	47.1 ± 0.6^b^
FRA	206 ± 5^c^	1.09 ± 0.01^b^	5.27 ± 0.02^c^	103 ± 3^b^	1012 ± 21^d^	576 ± 31^bc^	1107 ± 10^d^	50.3 ± 0.4^b^	36.4 ± 0.3^e^
EFR	195 ± 4^c^	1.06 ± 0.02^b^	5.40 ± 0.03^b^	98 ± 5^b^	840 ± 13^e^	419 ± 24^d^	933 ± 16^e^	33.4 ± 0.5^d^	40.1 ± 0.4^d^
GOR	200 ± 7^c^	1.08 ± 0.00^b^	5.41 ± 0.01^b^	100 ± 6^b^	805 ± 8^f^	383 ± 15^d^	897 ± 7^f^	29.6 ± 0.3^e^	42.0 ± 0.4^c^
LEC + FRA	264 ± 7^a^	0.87 ± 0.00^c^	3.31 ± 0.00^f^	132 ± 6^a^	1570 ± 23^a^	822 ± 35^a^	1721 ± 25^a^	74.1 ± 0.9^a^	32.5 ± 0.5^g^
LEC + EFR	249 ± 9^ab^	0.87 ± 0.00^c^	3.50 ± 0.02^e^	125 ± 4^a^	1311 ± 10^b^	613 ± 22^b^	1453 ± 13^b^	50.2 ± 0.7^b^	35.2 ± 0.3^f^
LEC + GOR	244 ± 5^b^	0.84 ± 0.01^d^	3.45 ± 0.02^e^	122 ± 5^a^	1222 ± 17^c^	513 ± 26^c^	1367 ± 17^c^	44.0 ± 0.8^c^	37.7 ± 0.6^e^

*In each column and in each section, means (± standard deviation) with different lowercase letters are significantly different (*P* < 0.05). ^A^Induction period (min), ^B^effectiveness, ^C^initiation rate constant (meq kg^−1^ min^−1^), ^D^ratio of the oxidation rate, ^E^antioxidant activity, ^F^the hydroperoxides concentration at the induction period (meq kg^−1^), ^G^propagation period (min), ^H^rate constant of the hydroperoxide formation in the propagation phase (min^−1^), ^I^rate constant of the hydroperoxide decomposition in the propagation phase (meq kg^−1^ min^−1^), ^J^maximum of the hydroperoxides concentration (meq kg^−1^), ^K^maximum rate of the hydroperoxide formation (meq kg^−1^ min^−1^), ^L^normalized form of the maximum rate (min^−1^), ^M^the hydroperoxides concentration at the turning point (meq kg^−1^); ^N^the occurrence time of the turning point (min), ^O^overall integration constant (kg meq^−1^), ^P^end time of the propagation phase (min), ^Q^the oxidation resistance (min/meq kg^−1^), ^R^carbonyl value in the turning point (μM g^−1^).

During the oxidation process, lipid systems can produce a variety of free radicals with different redox potentials (E_h_) such as alkyl (R^•^: 600 mV), alkoxyl (RO^•^: 1600 mV), peroxyl (ROO^•^: 1000 mV), and hydroxyl (^•^OH: 2320 mV)^29,30^. In the beginning of the oxidation process, the only pathway of ROOHs production is the conversion of R^•^ to ROO^•^ (due to its low E_h_) and its attack on the hydrogen attached to allylic or bis-allylic carbon^9^. Thus, Eq. (22) is the first and the most important defense barrier generated by antioxidant molecules. The *E* parameter led to results that indicated a higher efficiency of FRA in the hydrogen donating mechanism, compared to that in EFR and GOR (Table 1). However, as oxidation progressed, the pathway of reactions changed due to increasing ROOH molecules. These molecules attack the lipid substrate and contribute to the production of water, as evidenced by Eq. (23)^20^, thereby playing a key role in the oxidation process and in the performance of antioxidants:

23 $$ {\text{ROOH}}\, + \,{\text{RH}} \to {\text{RO}}^{ \bullet } \, + \,{\text{H}}_{{2}} {\text{O}}\, + \,{\text{R}}^{ \bullet } $$

As shown in Table 2, in the control sample, the amount of water increased in production during the peroxidation process, as attributeed to Eq. (23). However, in the presence of antioxidants, the amount of water production increased dramatically, compared to the control sample. This can be ascribed to the consumption of a part of antioxidant molecule in side reactions of the initiation chain, which produces water as shown in Eq. (24):

24 $$ {\text{AH}}\, + \,{\text{ROOH}} \to {\text{A}}^{ \bullet } \, + \,{\text{H}}_{{2}} {\text{O}}\, + \,{\text{RO}}^{ \bullet } $$

**Table 2:** Water content and reverse micelles size related to the stripped sunflower oil peroxidation (Control) containing lecithin (LEC), ferulic acid (FRA), ethyl ferulate (EFR), γ-oryzanol (GOR), and their combinations at 60 °C.

Sample	Water content (µg g^−1^)					Reverse micelles					
						Particle size (× 10^–2^) (nm)					Span^†^
	BIO	IP	AIP	PP	APP	BIO	IP	AIP	pp	APP	
Control	103 ± 15^Eb^*	137 ± 11.8^Dh^	540 ± 15^Cd^	940 ± 43^Bf^	1072 ± 56^Af^	0.94 ± 0.34^Ec^	6.27 ± 0.15^Cg^	1.21 ± 0.66^Df^	19.27 ± 0.15^Af^	12.95 ± 0.82^Bf^	1.05 ± 0.09^e^
LEC	199 ± 25^Ea^	245 ± 35^Dg^	719 ± 44^Cc^	1425 ± 49^Be^	1601 ± 41^Ae^	1.94 ± 0.10^Ea^	18.38 ± 1.04^Cd^	5.11 ± 0.33^Dc^	49.38 ± 0.60^Ac^	25.38 ± 1.27^Bc^	1.57 ± 0.12^c^
FRA	125 ± 12^Eb^	442 ± 21^Dd^	741 ± 28^Cc^	1898 ± 70^Bc^	2040 ± 62^Ac^	1.00 ± 0.22^Ec^	12.06 ± 0.46^Ce^	4.96 ± 0.40^Dc^	37.31 ± 1.04^Ad^	20.20 ± 0.90^Bd^	1.39 ± 0.04^c^
EFR	133 ± 18^Eb^	322 ± 4^De^	697 ± 19^Cc^	1711 ± 62^Bd^	1825 ± 37^Ad^	1.25 ± 0.18^Ebc^	10.27 ± 0.61^Cf^	3.24 ± 0.33^Dd^	32.24 ± 1.29^Ae^	17.44 ± 0.70^Be^	1.33 ± 0.04^d^
GOR	119 ± 14^Eb^	295 ± 1^Df^	715 ± 24^Cc^	1606 ± 56^Bd^	1752 ± 44^Ad^	1.51 ± 0.21^Eab^	9.62 ± 0.67^Cf^	2.15 ± 0.41^De^	29.88 ± 0.77^Ae^	16.21 ± 1.24^Be^	1.29 ± 0.06^d^
LEC + FRA	174 ± 27^Ea^	817 ± 24^Da^	1512 ± 25^Ca^	2980 ± 69^Ba^	3246 ± 95^Aa^	1.93 ± 0.25^Ea^	29.33 ± 0.68^Ca^	9.15 ± 0.58^Da^	58.12 ± 0.95^Aa^	32.75 ± 036^Ba^	1.84 ± 0.05^a^
LEC + EFR	186 ± 11^Ea^	709 ± 10^Db^	1405 ± 33^Cb^	2774 ± 71^Bb^	2955 ± 39^Ab^	1.77 ± 0.21^Ea^	25.91 ± 0.41^Cb^	7.03 ± 0.63^Db^	52.70 ± 1.23^Ab^	28.04 ± 1.00^Bb^	1.66 ± 0.07^ab^
LEC + GOR	161 ± 26^Ea^	631 ± 26^Dc^	1397 ± 40^Cb^	2635 ± 64^Bb^	2809 ± 77^Ab^	2.03 ± 0.36^Ea^	24.14 ± 0.50^Cc^	7.55 ± 0.70^Db^	49.17 ± 0.92^Ab^	26.12 ± 1.14^Bb^	1.60 ± 0.04^ab^

*In each row and in each section, averages (± standard deviation) with different uppercase letters are significantly different (*P* < 0.05). In each column, averages (± standard deviation) with different lowercase letters are significantly different (*P* < 0.05). *BIO* at the beginning of the oxidation, *IP* at the induction period, *AIP* after the induction period, *PP* at the propagation period, *APP* after the propagation period, Span = [(Dv0.9-Dv0.1)/Dv0.5] (Dv: represent particle sizes larger than 10, 50, and 90% of the population), ^†^the average spans of BIO, IP, AIP, PP, and APP.

A previous study indicated that GOR participates in this reaction^31^. Accordingly, it is a logical assumption that other antioxidants i.e. FRA and EFR can also take part in the Eq. (24) due to identical phenolic rings.

As shown in Table 1, the antioxidants were able to reduce the *k*_1_ and its derived index (i.e. *R*_or_), compared to non-inhibited peroxidation. The *R*_or_ is a symbol of electron transfer mechanism and it reflects variations in antioxidant radical (A^•^) performance^27,28^. Thus, the FRA with the lowest value of *R*_or_ showed the highest participation in quenching ROO^•^ by producing A-OOR. Generally, by integrating the results that pertained to the two mentioned mechanisms, the best antioxidant activity was observed in FRA, followed by EFR, and GOR (Table 1, *A* parameter). It is best to present a brief description of the achievements of LEC presence prior to discussing the behavior of antioxidants.

### Addition of LEC

The CMC of LEC is a criterion for introducing the maximum usable concentration beyond which LEC begins to self-aggregate and lose its effectiveness^22^. By the TCNQ method, the CMC of LEC was calculated as 12.71 mM and approximately a half of this amount was added to the bulk oil (to produce a heterogeneous bulk oil) so as to demonstrate its physicochemical properties. The addition of LEC to the functional environment of the antioxidants caused remarkable changes in the antioxidant performance (Table 1). As expected, LEC showed a limited antioxidant activity, but its synergistic effects were much more prominent (~ 65%). In the presence of LEC, a considerable change occurred in the mechanism of hydrogen donating of the antioxidants, which ultimately prolonged the IP. In an apparent contradiction, however, the presence of LEC caused a slight increase in the *k*_i_ compared to the absence of LEC (basic state). This indicates that the efficiency of the electron transfer mechanism decreased slightly and that the A^•^ participated in one or more of the propagation chain reactions, as shown in the following equations^28^:

25 $$ {\text{A}}^{ \bullet } \, + \,{\text{ROOH}} \to {\text{AH}}\, + \,{\text{ROO}}^{ \bullet } $$

26 $$ {\text{A}}^{ \bullet } \, + \,{\text{RH}} \to {\text{AH}}\, + \,{\text{R}}^{ \bullet } $$

27 $$ {\text{A}} - {\text{OOR}} \to {\text{AO}}^{ \bullet } \, + \,{\text{RO}}^{ \bullet } $$

28 $$ {\text{A}}^{ \bullet } \, + \,{\text{O}}_{{2}} \to {\text{AOO}}^{ \bullet } $$

The difference in the performance of antioxidants is likely due to the appropriate organization of the oxidative microreactors and the interaction of antioxidants with these structures, which is discussed in the following section.

### Interpretations of occurred events during the initiation phase

**Figure 3:**
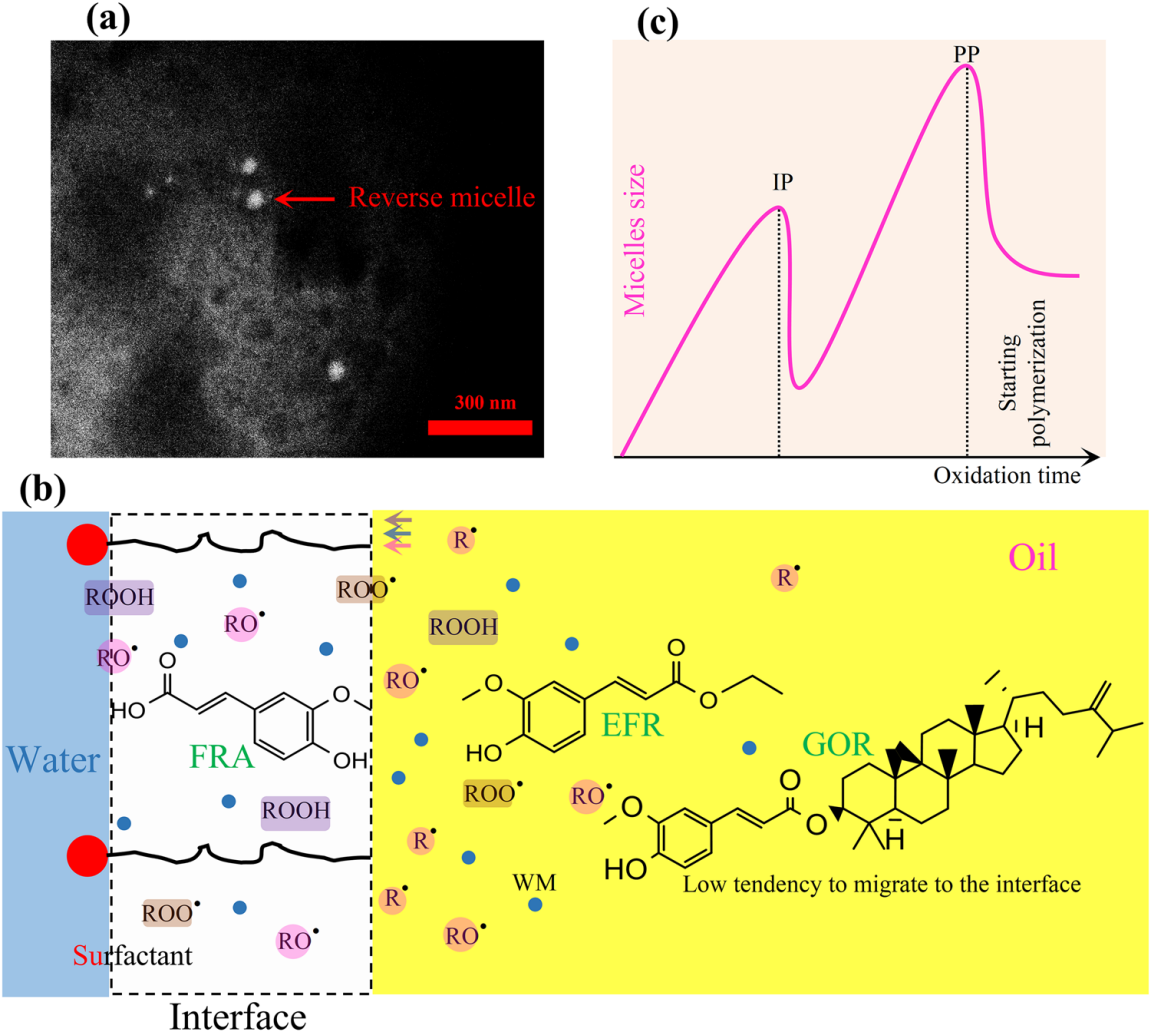
(**a**) TEM image of reverse micelles structures in the presence of γ-oryzanol during the induction period, (**b**) schematic figure of the cross-section of a reverse micelle produced during the oxidation process and a display of the dynamics of oxidation products as well as partitioning of the antioxidants under study in this structure (*EFR* ethyl ferulate, *FRA* ferulic acid, *GOR* γ-oryzanol, *R*^•^ alkyl radical, *RO*^•^ alkoxyl radical, *ROO*^•^ peroxyl radical, *ROOH* hydroperoxide, *WM* water molecule), (**c**) the changes in the trend of reverse micelles size during peroxidation of stripped sunflower oil at 60 °C.

For all samples, as the water content increased, the size of the reverse micelles kept increasing up to a point where the initiation phase ended (Table 2). In the presence of LEC, the size of reverse micelles increased more because of a greater reduction in interfacial tension. The effect of this behavior change is well observable in enhancing the micelles size at IP point (Table 2). Generally, the addition of LEC increased all kinetic parameters as shown in Fig. 2b. Such behaviors probably arose from an increase in the number of oxidation microreactors, as understood from Span changes (Table 2). Considering the migration of oxidation products to these structures, the accessibility of all oxidation active components can increase to each other. However, the main factor for the movement of antioxidants toward these structures is the hydrophilic–lipophilic balance, which is evaluated by log p (as a criterion of polarity)^9,12,16^. The values of log p were computed in the case of FRA (1.42), EFR (2.02) and GOR (10.12). Significant differences between these values can cause a difference in the interfacial performance of these compounds. The simultaneous presence of carboxyl and hydroxyl groups in the chemical structure of FRA can act as a potent driving force to move FRA toward the interface. However, the attachment of hydrophobic compounds to the carboxylic group (i.e. ethyl or phytosteryl) can increase the solubility of these compounds in the bulk oil. Therefore, there would be a decrease in the tendency of these compounds to migrate toward the interface, along with a decrease in their interfacial performance (Fig. 3a,b). The outcome of these events is a decrease in antioxidant activity. Obviously, the size of the attached hydrophobic group can play an important role in actualizing antioxidant activity^13^. However, our results showed that this decrease in efficiency is not uniform when there is an increase in the alkyl chain of the antioxidants.

**Figure 4:**
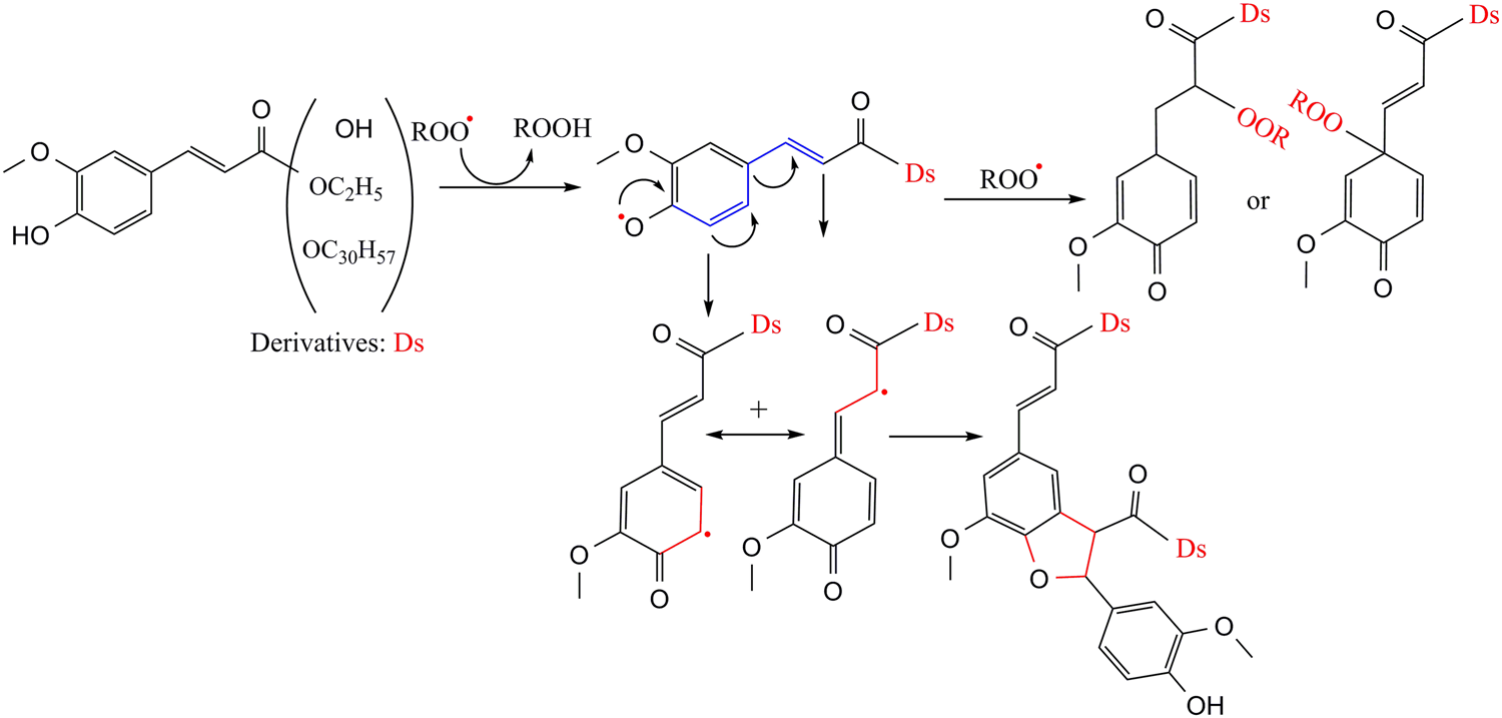
A proposed inhibitory mechanism of ferulic acid and its derivatives, as well as electron resonance delocalization during the oxidation process (as represented by the blue color), and the collision between two antioxidant radicals or peroxyl radical (ROO^•^) and antioxidant radical.

As mentioned earlier, the presence of LEC causes a remarkable increase in the *E* factor of antioxidants (~ 55%). Also, the *E* factor can enhance by increasing the antioxidant polarity. As shown in Fig. 2c, the growth coefficient in the presence of LEC was higher than that of the basic state (1.143 vs. 1.131). Thus, the synergy of these two factors (i.e. antioxidant polarity and presence of LEC) can considerably excite the participation of antioxidants in the mechanism of hydrogen donating. On the other hand, reducing the polarity of antioxidants increased their *R*_or_ factor, the growth coefficient of which was higher in the presence of LEC than its absence (0.881 vs. 0.868). Furthermore, the addition of LEC increased the *R*_or_ factor of antioxidants by ~ 4%, compared to the basic state. Therefore, antioxidants with lower polarity showed a higher degree of participation in Eqs. (25–28), while the participation was generally more severe in the presence of LEC. Such behavior likely originated from the partitioning of antioxidant molecules or of their radicals between bulk oil and microreactors of oxidation. The behavior is probably a manifestation of their polarity. The ROOHs of sunflower oil are likely to move to the interface because of their higher polarity and a greater driving force which, in turn, is caused by the presence of at least two oxygen molecules on their allylic and/or *bi-*allylic carbon^32,33^. Therefore, the effective collisions decrease between less polar antioxidants and intermediate components of oxidation. As shown in Fig. 4, the inhibitory pathways of the antioxidants under study can be different. In fact, the relatively high energy of bound dissociation (–OH)^11^ can make FRA and its derivatives unable to quench the R^•^, so that their only pathway in demonstrating antioxidant activity is the reaction with ROO^•^. Considering that the ROO^•^ is necessarily located in the interface, a lower level of access to these radicals ensues among antioxidants which lower polarity. Accordingly, the access of A^•^ to target free radicals is reduced as well. Thus, A^•^ must either participate in the side reactions of the propagation chain or in the neutral reaction of the termination chain, i.e. the collision between the two radicals (A^•^ + A^•^ $\to$ product)^34^.

### Key role of water in the oxidation process

As can be deduced from Table 2, the increase in the size and number of reverse micelles is in parallel with the amount of water produced in the system. These water molecules have a high tendency to attach to the hydrophilic head of LEC for a decrease in interfacial tension^34^. Therefore, the formation of reverse micelles likely accelerates by creating preliminary cores arising from water production. Obviously, the existing antioxidants in the environment will have a better chance to be deployed in the interface (where free radicals are located) by increasing the number of microreactors^20,33^. As shown in Fig. 2d, a desirable correlation was found between the amount of water being produced in the initiation phase of oxidation and the *E* factor. This can prove that the hydrogen donating mechanism of antioxidants becomes more active by increasing the water content.

As oxidation progressed, the production of water and ROOHs increased uniformly, but the migration rate of water molecules into the core of the micelle probably occurred faster than the ROOHs to the interface. This difference in rate can be explained by the small size of water molecules and the high driving force. These events probably caused the core of the micelle to grow faster, so that existing surfactants and the resultant ROOHs became insufficient to cover this increase in volume. Thus, the reverse micelles disintegrated. The result of these events can be the transition the initiation phase to the propagation.

### Kinetic parameters of propagation and termination phases

As listed in Table 2, the size of the reverse micelles significantly reduced after the IP point (AIP), and a relative physical stability was probably established in the system. However, the chemical reactions that happened thereafter were assumed to be completely different. Homolytic decomposition (Eq. 29) is one of the most important reactions that occurs during lipid peroxidation^1,6^:

29 $$ {\text{ROOH}} \to^{ \bullet } {\text{OH}}\, + \,{\text{RO}}^{ \bullet } $$

The products of this reaction are very important. Given that the oxygen in the RO^•^ usually mounts onto the *bis*-allylic carbon, the β-cleavage reaction occurs definitely because this type of radical is instable. It causes the surface-active compounds to be produced at a higher level than in the initiation phase^1^. On the other hand, the hydroxyl radical that is produced according to Eq. (29) can attack each compound due to its high E_h_ and, thus, can separate its H^29,30^.This radical causes water production by receiving hydrogen (Eq. 30), which provides conditions to increase the micelles size.

30 $$ {\text{HO}}^{ \bullet } \, + \,{\text{RH }}\left( {\text{or any other similar compounds}} \right) \to {\text{H}}_{{2}} {\text{O}}\, + \,{\text{R}}^{ \bullet } $$

The collision of accumulated molecules of ROOH with each other is another reaction that occurs during the propagation phase. The product of this reaction, known as the bimolecular reaction, is water as shown in the following equation^20^:

31 $$ {\text{2ROOH}} \to {\text{ROO}}^{ \bullet } + {\text{H}}_{{2}} {\text{O}} + {\text{RO}}^{ \bullet } $$

The water molecules produced by Eqs. (30) and (31) usually play a very important role in the events that occur during the propagation phase.

A considerable difference was observed between the duration of PP in the inhibited peroxidation, compared to the non-inhibited condition (Table 1). Considering that the lipid substrate is composed of only pure triacylglycerols of sunflower oil, each event is a direct result of the antioxidant performance which is added to the system. Therefore, it is concluded that antioxidant molecules are not entirely consumed during the initiation phase of oxidation and their remnants indicate some antioxidant activity during the propagation phase. Moreover, the addition of LEC caused a tangible increase in the duration of PP, which is likely due to the physical role of this compound in inhibiting oxidation reactions. The onset of the propagation phase is associated with the regeneration of the reverse micelles and with an increase in their size through time (Table 2), meaning that the physical events in the initiation phase of oxidation are likely to reoccur in the propagation phase (Fig. 3c). However, at this stage, the size of reverse micelles increased considerably due to the addition of a large volume of surface-active agents produced by the oxidation process. Obviously, all radicals have a strong tendency to migrate toward the reverse micelles^33^, and the formation of reverse micelles is supported by the presence of LEC. Thus, the reverse micelles tend to multiply in number and enlarge, compared to the basic state (Table 2). As a result, the LEC causes a delay in the secondary breaking point of the reverse micelles, i.e. ROOH_max_ (Tables 1, 2). In fact, the ROOH_max_ is a secondary CMC in the peroxidation of oils, as it occurs precisely at the end of the propagation phase (Table 2). Through these events, the PP duration is prolonged. The results indicated that adding LEC reduced the amounts of *k*_f_ and *k*_d_ remarkably, compared to the control sample (Table 1) (*k*_c_: 2.87 vs. 1.69; *k*_d_: 1.35 vs. 0.64). These results confirm the physical role of LEC in the inhibition of oxidation reactions in the propagation phase.

The results of the maximum rate of ROOHs formation (M_R_) showed that samples which had been treated with the antioxidant, compared to untreated samples, reached a lower rate at the turning point (Table 1). This can be attributed to the remaining molecules of the antioxidant in the propagation phase that may act as a barrier to the actualization of a maximum rate. Meanwhile, it should be considered that the maximum rate occurs within a specified concentration range of ROOH, i.e. ${{[\mathrm{ROOH}]}_{\mathrm{M}}}_{\mathrm{R}}$ (Table 1). In the presence and absence of LEC, the average of these concentrations are equal to 127.51 ± 4.94 meq kg^−1^ (LEC + (FRA + LEC) + (EFR + LEC) + (GOR + LEC)) and 101.98 ± 3.90 meq kg^−1^ (Control + FRA + EFR + GOR), respectively.

${{\mathrm{N}}_{\mathrm{M}}}_{\mathrm{R}}$ is a symbol of lipid resistance against propagation chain reactions, in which the lower values of this criterion indicate a higher resistance of the system^5^. The results showed that adding LEC significantly reduced this parameter (Table 1). The end time of the propagation phase (Et_PP_) indicated that the highest and the lowest time in the inhibited peroxidation pertained to FRA + LEC and GOR, respectively (1721 vs. 897 min) (Supplementary Information 1).

Carbonyl compounds, including ketones and aldehydes, are considered as a very important group of secondary oxidation products that cause rancid and undesirable flavors in the lipid systems^26^. The amount of carbonyl compounds at the turning point (${\mathrm{CV}}_{{\mathrm{M}}_{\mathrm{R}}}$) indicated that this index has a reverse relationship with the antioxidant activity (Table 1). Considering that the inhibitory activity of antioxidants under study showed a desirable correlation with the water content of the system, it can be concluded that the amount of the produced carbonyl compounds has an indirect and negative dependence on the produced water content in the lipid system.

**Figure 5:**
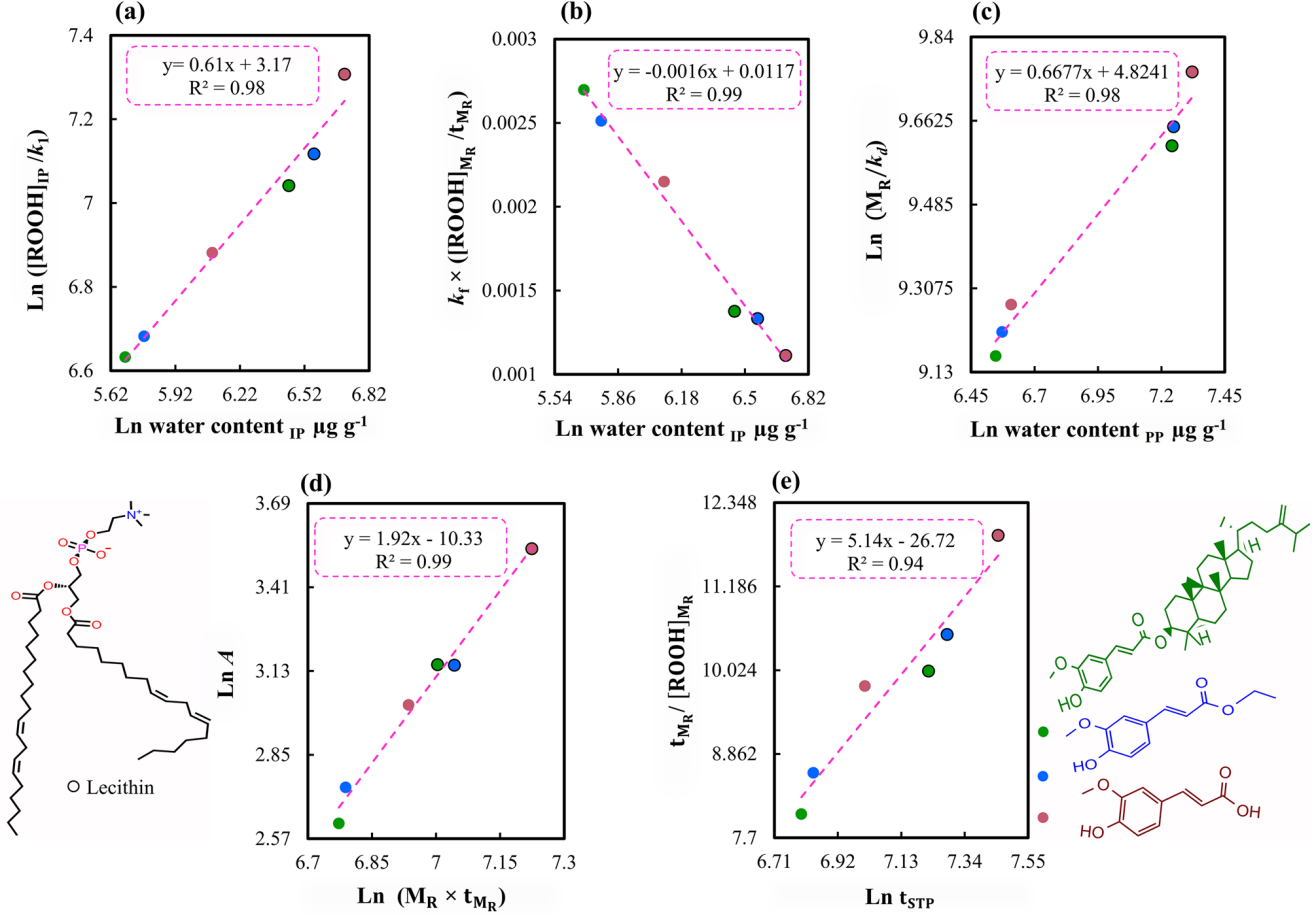
Relationships between various kinetic parameters in the peroxidation of stripped sunflower oil containing lecithin, ferulic acid, ethyl ferulate, γ-oryzanol and their combinations at 60 °C. *A* antioxidant activity, *Et*_*PP*_ end time of the termination phase or *t*_*STP*_, *k*_*f*_ rate constant of hydroperoxides (ROOHs) formation at the propagation phase, *k*_*d*_ rate constant of ROOHs decomposition at the propagation phase, *k*_*1*_ rate constant of the initiation phase, *M*_*R*_ maximum rate of ROOHs formation in the propagation phase, *[ROOH]*_*IP*_ ROOHs concentration at IP point, ${\left[ROOH\right]}_{{M}_{R}}$ ROOHs concentration at the point of the maximum rate of ROOHs formation (or turning point), ${t}_{{M}_{R}}$ occurrence time of the maximum rate of ROOHs formation (or turning point), *water content*_*IP*_ water content at the IP point, *water content*_*PP*_ water content at the PP point.

Most oxidation products that are produced over time convert to other products due to their high reactivity and, thus, the trends of their production can fluctuate frequently. The water content is one of the most stable oxidation indices that can be produced during the oxidation process^20^. The results showed that this parameter has various linear relationships with some oxidation parameters of the initiation phase or of the propagation phase. For example, Fig. 5a shows the relationship between one of the key parameters, namely the ratio of the maximum achievable concentration of ROOHs in the initiation phase to its rate constant (ROOH_IP_/*k*_*1*_) and the water content at the IP point. This relationship shows that increasing the water content can significantly reduce the oil peroxidation rate at the initiation phase. Interestingly, the amount of water at the IP point can even change the overall trend of the oxidation process at the propagation phase. For example, Fig. 5b shows that multiplying the *k*_f_ with the ratio of the coordinates at the turning point (${{[\mathrm{ROOH}]}_{\mathrm{M}}}_{\mathrm{R}}$/${{\mathrm{t}}_{\mathrm{M}}}_{\mathrm{R}}$) correlates with the water content at the IP point. In addition, the water content being produced during the propagation phase effectively enhances the ratio of the maximum rate of ROOHs formation to the rate of their decomposition (M_R_/k_d_) (Fig. 5c). This effect can be attributed to the overall reduction of the rate constant of ROOHs decomposition by increasing water content. Considering that the number of oxidation microreactors increases by increasing the water content, many sites would exist to enhance the overall capacity of receiving ROOHs. Thus, the probability of effective collisions between ROOHs decreases and, accordingly, their decomposition occurs more slowly.

Generally, the results showed that the said events have several effects during the initiation phase of the oxidation. They could considerably affect the parameters in relation to the turning point. On the other hand, changing the position of time or concentration of the turning point can also affect the end time of the propagation phase. For instance, Fig. 5d shows a linear relationship between the antioxidant activity and the result of multiplying the maximum rate of ROOHs production with the occurrence time of the turning point (M_R_ × ${{\mathrm{t}}_{\mathrm{M}}}_{\mathrm{R}}$). Given that the M_R_ of the antioxidants did not change much (Table 1), it can be concluded that improving the efficiency of antioxidants remarkably increases the occurrence time of the turning point. Figure 5e shows a linear relationship between the time/concentration coordinates of the turning point and the end time of the propagation phase. This result suggests that the delay in achieving the turning point can considerably increase the propagation period. The turning point in the middle of the propagation phase appears to be the point where the antioxidant activity becomes zero. In general, the set of Fig. 5 proves that all of the events that occurred in the lipid oxidation process were interconnected like an intertwined chain.

## Conclusion

The present research can considerably change the prospects for practical applications of relatively polar antioxidants. The paradigm selected in this study is a feedback about the use of antioxidants that are likely to migrate to the water–oil interface, despite having sufficient solubility in oil environments. Thus, a fundamental change can probably take place in using these antioxidants, so that adding specialized surfactants to oil environments in the presence of these antioxidants would remarkably increase their efficiency. Irrespective of the macro objectives of this project, our results revealed facts that had previously been less sought for in research. One of the most important achievements of this study was the identification of key roles of water production during the process of lipid oxidation, as a major, basic element in directing this process. Another achievement of this research explained in detail how physicochemical events occur during oil oxidation and how their role can assist in the evolution of this process. Our understanding of fundamental facts in relation to the oxidation process is still insignificant, although the present study can be an inspiring step forward.

## Supplementary Information

Supplementary Information 1.

## Abbreviations

*A*: Antioxidant activity
a: Integration constant of sigmoidal model
A^•^: Antioxidant radical
AH: Antioxidant molecule
AIP: After IP point
CMC: Critical micellar concentration
*E*: Effectiveness of antioxidant
EFR: Ethyl ferulate
E_h_: Redox potential
Et_PP_: End time of the termination phase
FRA: Ferulic acid
GOR: γ-Oryzanol
H: Hydrogen
IP: Induction period
*k*_f_: Rate constant of hydroperoxides formation at the propagation phase
*k*_d_: Rate constant of hydroperoxides decomposition at the propagation phase
*k*_1_: Rate constant of the initiation phase
LEC: Lecithin
Log P: Partition coefficient
M_R_: Maximum rate of hydroperoxides formation in the propagation phase
${{\mathrm{N}}_{\mathrm{M}}}_{\mathrm{R}}$: Normalized form of maximum rate of hydroperoxides formation in the propagation phase
PP: Propagation period
OH: Hydroxyl group
^•^OH: Hydroxyl radical
R^•^: Alkyl radical
RH: Lipid reactant
RO^•^: Alkoxyl radical
ROO^•^: Peroxyl radical
ROOHs: Hydroperoxide(s)
ROOH_IP_: The hydroperoxides concentration at IP point
[ROOH]_max_: Maximum concentration of the produced hydroperoxides
${\left[\mathrm{ROOH}\right]}_{{\mathrm{M}}_{\mathrm{R}}}$: The hydroperoxides concentration in the point of maximum rate of hydroperoxides formation (or turning point)
R_or_: Ratio of oxidation rate
TCNQ: Tetracyanoquinodimethane
${\mathrm{t}}_{{\mathrm{M}}_{\mathrm{R}}}$: Occurrence time of maximum rate of hydroperoxides formation
