# Supplementary figures and images for: Investigating relationship between water production and interfacial activity of γ-oryzanol, ethyl ferulate, and ferulic acid during peroxidation of bulk oil
Source: Sci Rep. 2021 Aug 23;11:17026. doi: 10.1038/s41598-021-96439-9 (PMC8382700; doi:10.1038/s41598-021-96439-9)

**
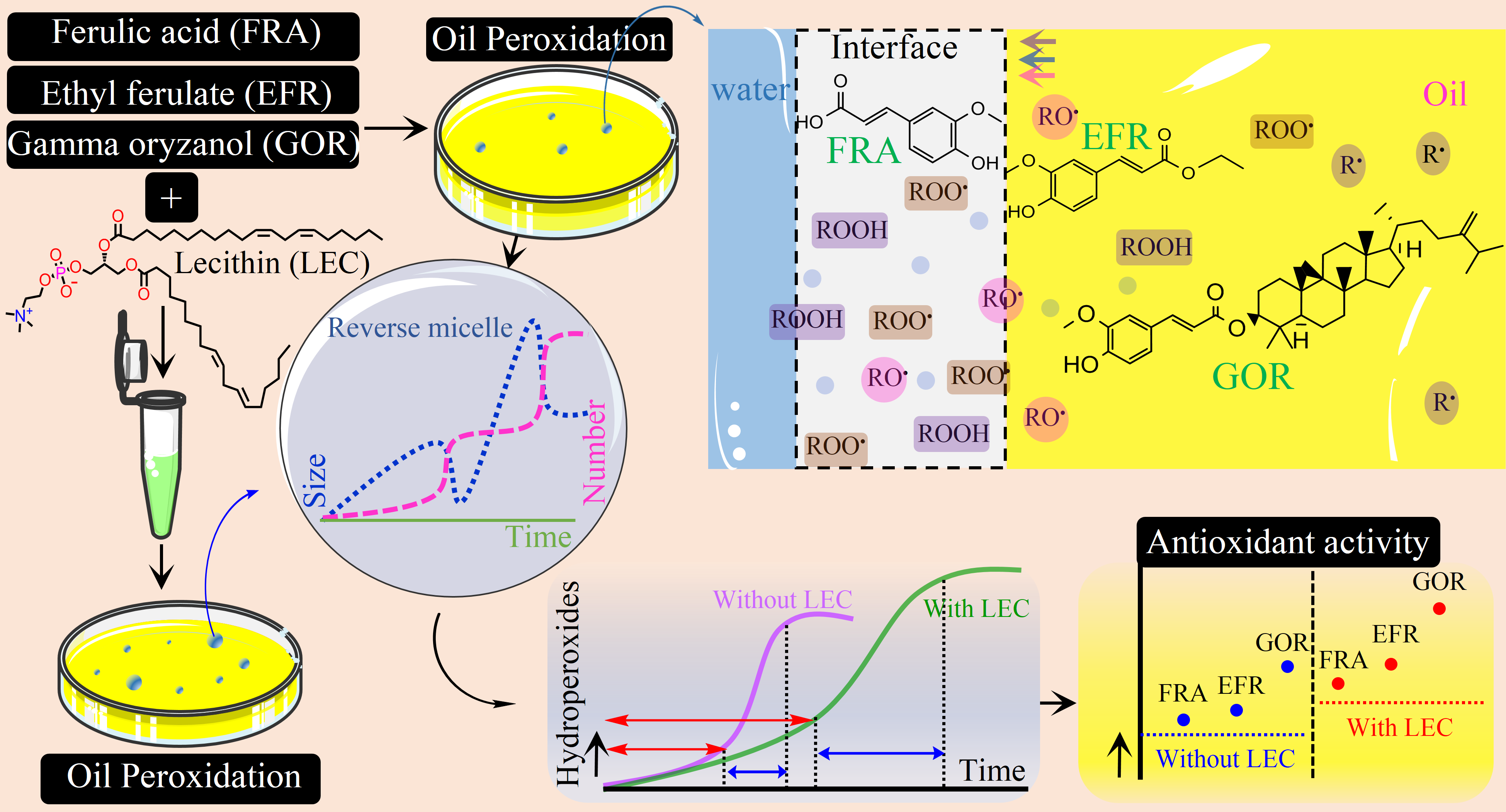
**

**Supplementary Figure 1.** Schematic diagram of research.

Supplement: Supplementary file 1 — Supplementary Information 1. [file 41598_2021_96439_MOESM1_ESM.docx]
